# Antioxidant Properties and Beneficial Cardiovascular Effects of a Natural Extract of Pomegranate in Healthy Volunteers: A Randomized Preliminary Single-Blind Controlled Study

**DOI:** 10.3390/antiox11112124

**Published:** 2022-10-28

**Authors:** Emad A. S. Al-Dujaili, Ciara Casey, Angela Stockton

**Affiliations:** 1 Centre for Cardiovascular Science, Queen’s Medical Research Institute, University of Edinburgh, 47 Little France Crescent, Edinburgh EH16 4TJ, Scotland, UK; 2 Dietetics, Nutrition and Biological Sciences, Queen Margaret University, Edinburgh EH21 6UU, Scotland, UK

**Keywords:** pomegranate, cardiovascular disease, blood pressure, polyphenols, lipid peroxidation, antioxidant capacity, cortisol, bioelectrical impedance

## Abstract

Pomegranates are known to possess anti-hypertensive, anti-atherogenic and cardioprotective effects mainly due to their pleiotropic effects on various cellular pathways, especially those triggered by oxidative stress. The aim of this study was to investigate the effect of natural standardized pomegranate (PE) extract on cardiovascular risk factors in 24 healthy volunteers who participated in a randomized, single-blind placebo-controlled study. There were 12 subjects in the PE group and 12 in the placebo group. Variables were measured at baseline and after 14 and 28 days of supplementation are anthropometry, BP, pulse wave velocity, fat and lean body mass, salivary and urinary cortisol, and cortisone, total phenolics, antioxidant capacity and lipid peroxidation. Urinary total phenolics excretion and antioxidant capacity were significantly increased after 14 and 28 days of PE intake. At day 28, there were also statistically significant decreases in systolic and diastolic blood pressure (BP), pulse wave velocity, body fat and fat mass, as well as an increase in lean body mass. Significant changes in the placebo group were not found. Glucocorticoid levels showed a significant decrease in saliva cortisol at day 28 (morning) in the PE group, and cortisol/cortisone ratio was significantly decreased following 28 days of PE intake at morning, noon, and evening. Urine free cortisol was significantly reduced at day 14. These findings suggest that pomegranate extract intake may improve antioxidant and oxidative stress status and play a beneficial role in the attenuation of some cardiovascular risk factors. Future studies should concentrate on overweight and older people.

## 1. Introduction

Cardiovascular diseases (CVDs) are the leading cause of death globally [1]. In the 2019 Global Burden of Disease study of 369 diseases in 204 countries and territories, ischemic heart disease and stroke were the top rank causes of disability-adjusted life years (DALYs) in adult age groups [2]. In Europe, CVD accounts for 45% of all deaths and is responsible for 23% of all DALYs lost [3]. In the European Society of Cardiology (ESC) member countries, ischemic heart disease and stroke accounted for 82% of DALYs [3]. There is extensive evidence of the effects of potentially modifiable risk factors on major cardiovascular events and mortality, particularly behavioral and metabolic factors including tobacco smoking, alcohol consumption, unhealthy diets, physical inactivity, high sodium intake, dyslipidemia, hypertension, type 2 diabetes mellitus, and overweight/obesity [4,5,6]. Glucocorticoids and mineralocorticoids have been associated with the progression and increased incidence of CVD diseases [7,8]. CVD is multifactorial and involves a complex interplay between modifiable and fixed (genotype, age, menopausal status, gender) causative factors [9], with oxidative stress, reactive oxygen species, inflammatory mediators and free radical damage playing a central role in the heterogeneous pathophysiological mechanisms underlying endothelial dysfunction and atherosclerotic plaque formation [10,11,12,13,14].

Promoting healthy dietary composition may prevent many primary and secondary cardiovascular events [15,16]. Diets high in fruits, vegetables, whole grains, nuts, and legumes; moderate in low-fat dairy and seafood; and low in processed meats, sugar-sweetened beverages, refined grains, and sodium are recommended [16]. High intake of fruit and vegetables rich in phytochemicals comprising of carotenoids and polyphenols such as ellagitannins, resveratrol, isothiocyanates, flavonoids and organosulfur compounds play an important role in reducing the risks of developing non- communicable diseases including cancer and CVD [17,18,19].

Biophenol-rich products obtained from pomegranate (*Punica granatum* L.) have been a focus of increasing interest in the potential health benefits due to their pleiotropic effects affecting various cellular pathways, especially those triggered by oxidative stress [20,21]. The therapeutic potential of pomegranate has been used for centuries in many cultures for its multipotent properties in the prevention and treatment of different health disorders, and nowadays a wide variety of pomegranate-based components have been developed as functional food supplements [22,23,24]. Pomegranate has a remarkable antioxidant activity due to its high content in polyphenols and flavonoids, including ellagitannins, anthocyanins, gallic acid derivatives, punicalagin, punicalin, pedunculagin, ellagic acid, phenolic acids and other complex flavonoids. When compared to a variety of natural-derived antioxidants, pomegranate polyphenols are thought to demonstrate superior effects regarding protecting lipoproteins from oxidation thereby playing a fundamental role in the attenuation of atherosclerotic development and subsequent cardiovascular events [25,26,27,28]. It has been shown that consumption of pomegranate extracts and juice have been associated with beneficial effects on blood pressure and cardiovascular health [29,30,31]. However, data on the anti-hypertensive, anti-atherogenic and cardioprotective effects of pomegranate preparations in human studies are still limited [24,25,27,31]. Therefore, a study was conducted in healthy volunteers, the aim of which was to add evidence of the effect of a standardized pomegranate extract on cardiovascular risk factors, including anthropometric variables, blood pressure and steroid hormones. The antioxidant capacity, lipid peroxidation and bioavailability of pomegranate polyphenols were also investigated.

## 2. Materials and Methods

### 2.1. Design and Participants

This was a randomized, single-blind, and placebo-controlled study conducted in healthy volunteers. The duration of the study was 28 days. This study was part of a larger trial that has been registered with ClinicalTrials.gov as NCT02005939. Volunteers were recruited through word-of-mouth at Queen Margaret University, Edinburgh, UK, and an e-mail moderator advertisement, which was sent to all students and staff within the university. The study was open to men and women from 18 to 65 years of age, with a body mass index (BMI) between 18 and 34 kg/m^2^. Individuals with CVD, chronic diseases, hypertension or receiving anti-hypertensive medication or any other regular drug treatment, type 2 diabetes mellitus, asthma, thyroid diseases, and eating disorders were excluded. Pregnant and breastfeeding women, subjects with allergy to pomegranate and those taking supplements that may interfere with the study products were also excluded. Participants were asked to refrain from sterenous extercise and alcohol consumption at least 24 h prior to all 3 study visits. The study protocol was approved by the Institutional Review Board of Queen Margaret University (code13000065/Master DM009/Pomegranate extract/DNBS/QMU) and written informed consent was obtained from all participants. All data were anonimyzed, coded and securely stored on a password-protected database.

### 2.2. Treatment

Participants were provided a lifestyle questionnaire to determine their eligibility, and were asked to maintain their usual diet and exercise regimens throughout the intervention, as well as to reduce their intake of foods rich in polyphenols. The study consisted of a daily oral intake of pomegranate (PE) capsules or placebo for 28 consecutive days. Participants were randomly assigned to receive either PE or placebo by a computer-generated random number list prepared by an independent investigator. Each PE capsule contained a standardized extract of whole pomegranate fruit obtained by an eco-friendly process (Pomanox^®^ P30, Euromed S.A., Barcelona, Spain), with the following composition: 210 mg punicalagin (the recommended daily intake to provide the beneficial effects of these antioxidants) and 328 mg total polyphenols, including other pomegranate polyphenols (e.g., flavonoids, ellagic acid and 0.37 mg anthocyanins). Placebo capsules contained maltodextrin, and both PE and placebo capsules were identical in appearance weighing 1.08 g each containing 6.52 kcal per capsule.

### 2.3. Study Procedures

**Figure 1:**
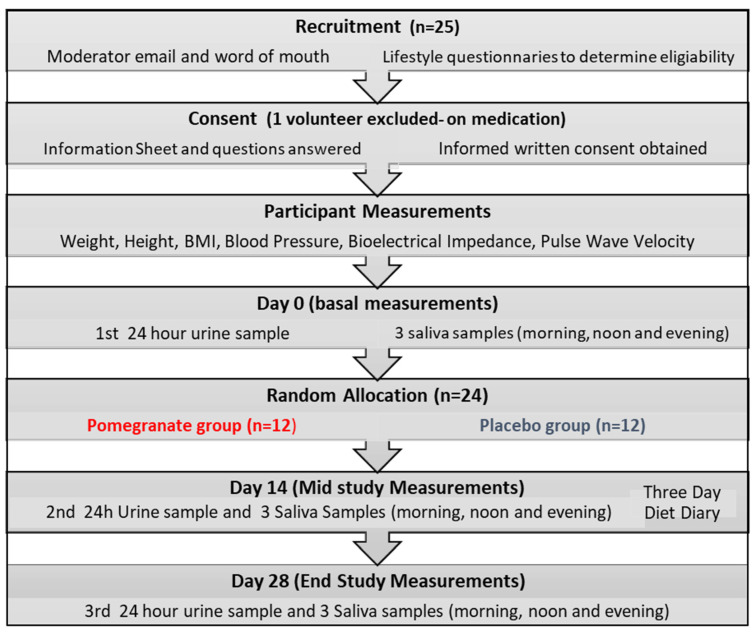
Timeline of the study protocol.

Prior to randomization, eligible subjects attended a 30-min session in the food research laboratory of the study center where baseline measurements were obtained, including body weight, height, BMI, blood pressure (BP), pulse wave velocity and bioelectrical impedance. The study included three visits at pre-treatment/baseline (visit 0) and at 14 days (visit 14) and 28 days (visit 28 end of study) after starting consumption of PE or placebo. At the study visits anthropometric measurements were repeated and saliva and 24-h urine samples were obtained for assessment of glucocorticoids, total phenolics, antioxidant capacity, lipid peroxidation and bioavailability of PE polyphenols. Compliance with the study product was measured by assessing total phenolic content of 24-h urine samples which were collected on day 0, 14 and 28 (see Figure 1).

#### 2.3.1. Anthropometric, Body Composition and Aortic Stiffness Measurements

Height was measured using a wall mounted stadiometer, and body weight using a calibrated Salter weighing scale. Three measurements were taken and the average was calculated. Body composition was assessed by bioelectrical impedance analysis (BIA) (Bodystat) and measurements were taken in triplicate from the right side of the participant [32]. Blood pressure (BP) was measured using an automatic BP monitor (UA767 Plus 30, A & D Medical, Tokyo, Japan) and the average of three readings taken at 2-min intervals was used. Hypertension was defined at 140/90 mmHg or greater. Continuous 24-h blood pressure measurements should have been used instead [33], however this study was designed exclusively to explore the antioxidant properties of PE to reduce non-compliance. Supine carotid to femoral pulse wave velocity (cfPWV) was measured using the Vicorder (Skidmore Medical, Bristol, UK) device; three measurements were taken over 5 cardiac cycles and the mean value was taken for analysis [34]. Results of cfPWV were expressed as meters per second (m/s).

#### 2.3.2. Collection of Saliva and Urine Samples

Three saliva samples were required on days 0, 14 and 28. Samples were collected by spitting (approximately 2–3 mL) into a 5 mL sterile container prior to each meal (breakfast, lunch, and dinner) over a period of one day. By the end of the intervention participants should have a total of 9 saliva samples which were stored in a dedicated freezer at −20 °C. One 24-h urine sample was also required on days 0, 14 and 28. Urine was collected in a 2 L container and total volume was weighted using calibrated weighting scales. After thorough mixing, 40 mL of urine was divided and transferred into two sterile containers. The empty urine container was reweighed, and the total volume of urine was recorded. All 20 mL urine samples were then frozen at −20 °C.

#### 2.3.3. Determination of Glucocorticoids

Cortisol and cortisone levels were estimated in both urine and saliva samples using a highly sensitive enzyme-linked immunoassay (ELISA) technique previously described by Al-Dujaili et al. [35]. Saliva and urine samples were thawed, vortexed for approximately 2 min prior to being transferred to Eppendorf tubes for centrifugation at 4800× *g* for 10 min (urine samples required less time, approximately 3 min) to remove any impurities. An aliquot of 250 µL of saliva or 100 µL of urine was then pipetted into new Eppendorf tubes and up to 1.5 mL of diethyl ether was added. Using a multi tube vortexer, the samples were vortexed for 10 min and then frozen at −80 °C for a further 10 min to allow the separation of the aqueous fraction from the ether fraction; with the latter being decanted into Pyrex glass tubes. The ether was evaporated at 40 °C under nitrogen and reconstituted with 250 µL and 500 µL of ELISA assay buffer for saliva and urine samples, respectively. For the ELISA assay, standards were prepared by serial dilutions of cortisone and cortisol stock solutions. Cortisone standards were 0, 0.5, 1.0, 2.5, 10 and 50 ng/mL while cortisol standards were 0, 2.5, 10, 50, 250 and 1000 ng/mL. Individual 96 well microplates were coated with cortisol or cortisone-BSA conjugates and were allowed to incubate overnight at 4 °C. The plates were washed and 200 µL of block buffer was added, incubated at 37 °C for 1 h. Standards and samples (50 µL) were added to the plates followed by 100 µL of steroid antibody and plates incubated at room temperature for 2 h. Enzyme reagent was added to the wells (HRP conjugated to anti-rabbit for cortisone and anti-sheep for cortisol) and plates incubated another hour. Substrate reagent (100 µL) was added followed by a 15-min incubation for color development. The reaction was then stopped by adding 50 µL stop solution and the color was read by an ELISA reader at 450 nm. Cortison and cortisone were expressed as ng/mL in saliva and as nmol/day in urine.

#### 2.3.4. Total Phenolic Products

Total phenolic products in urine were determined using the method described by Singleton and Rossi [36]. Gallic acid standards 50, 100, 200, 300, 400, and 500 mg/L were prepared. In Pyrex tubes, 50 µL of standard or 50 µL of a 1:5 dilution of urine samples was added to 2.5 mL of a 1:10 dilution of Folin and Ciocalteau reagent and 45 µL deionized water. After 5 min, 1.6 mL sodium carbonate solution was then added, and the reaction mixture was allowed to stand in darkness at room temperature for 2 h. The blue color generated from reducing yellow heteropoly-phosphotunstate-molybdate anions was read against a water blank at 765 nm on the spectrometer. Results were expressed as mg of gallic acid equivalent (GAE)/day. Furthermore, the bioavailability of PE polyphenols were analyzed in 24-h urine samples from a randomly selected subject treated with PE at the James Hutton Institute (Dundee, UK) using liquid chromatography-mass spectometry (LC-MS) to identify urinary metabolites.

#### 2.3.5. Antioxidant Capacity and Lipid Peroxidation

The antioxidant capacity in urine samples was measured using the ferric reducing antioxidant power (FRAP) assay [37]. Reagents were prepared as directed and standards were prepared by diluting ferrous sulphate with deionized water at concentrations of 0.1, 0.2, 0.4, 0.5, 0.6, 0.8 and 1.0 mmol/L. Using a 96 well plate, 10 µL of standard or 10 µL of a 1:5 urine dilution was pipetted into individual wells. FRAP solution (250 µL) was added and the plate was incubated at 37 °C for 5 min. The plate was then read at 600 nm using a microplate reader [38]. Results were expressed as mmoL of ferrous sulphate (Fe^+2^)/day.

Lipid peroxidation in urine samples was measured using the thiobarbituric acid reactive substances (TBARS) assay, assessing malondialdehyde (MDA) as end product of lipid peroxidation [39]. Reagents including a 150 mM Tris-HCL buffer, 1.5 mM ascorbic acid and ferrous sulphate, 30% TCA and 0.75% thiobarbituric acid (TBA) solution were prepared. Standards were prepared from a stock MDA solution of tertramethoxy-propane 1 mM (TMP) in deionized water and the following concentrations were used: 100, 50, 25, 10, 5, 2.5 and 0.625 µmol/L. In 2 mL Eppendorf tubes, 0.1 mL of standard or 0.1 mL of urine, 0.2 mL Tris’s buffer and 0.1 mL ascorbic acid ferrous sulphate solution were added and incubated in a water bath at 37 °C for 15 min. Then, 0.4 mL of TCA and 0.8 mL of TBA were added to the tubes, covered with aluminium foil, and further incubated in a water bath at 98–99 °C for 15 min. Once cooled, the tubes were centrifuged at 4000× *g* for 10 min. The supernatant was decanted into cuvettes and read on the spectrometer at 532 nm. Results were expressed as µmol MDA/day.

### 2.4. Assessment of Nutritional Intake

On day 14 of treatment, participants were requested to complete a 3-day diet diary on two midweek days and one weekend day [40]. The 3-day food diaries were analyzed with the NetWISP analysis program (version 4.0) (Tinuviel Software, Llanfechell, Anglesey, UK), and energy, protein, fat, and carbohydrate intake were determined. Participants were blind to what the food diary was assessing.

### 2.5. Statsitical Analysis

Continuous data expressed as mean and standard deviation (SD). Independent paired t-tests were used to assess differences in pre-treatment variables in the PE and placeblo groups for body weight, BMI, systolic (SBP) and diastolic (DBP) blood pressure, fat mass, fat free mass, lean mass, salivary and urinary cortisol and cortisone, cortisol to cortisone ratio, and antioxidant capacity (total polyphenols, TBARS and FRAP). The relationship between the antioxidant capacity and total phenolic products were assessed using Pearson’s correlation coefficient (*r*). Differences between post-treatment and pre-treatment variables in the PE and placebo groups were analyzed using analysis of covariance (ANCOVA), with post-treatment measures as the dependent variable and treatment group (PE or placebo) as the fixed independent factor and pre-treatment/baseline measurements as the covariates. The Bonferroni’s correction was applied to account for multiple testing. Statistical significance was set at *p* ≤ 0.05. Data were analyzed using SPSS for Windows^®^ version 21.0 (IBM Corp 2012, New York, NY, USA).

## 3. Results

### 3.1. Study Population

A total 24 subjects (10 men, 14 women) aged between 20 and 27 years met the inclusion criteria and gave written consent to participate in the study. Twelve subjects were assigned to the PE group and 12 to the placebo group. As shown in Table 1, there were no statistically significant differences in baseline/pre-treatment (day 0) anthropometric, physiological, hormone, and antioxidant capacity variables.

### 3.2. Changes of Anthropometric, Body Composition, BP and PWV

Weight, BMI, and body composition were assessed on days 0 (pre-treatment) and 14 and 28 (post-treatment). When comparing these measurements at these time points, no significant changes in weight and BMI were observed in either the PE or the placebo group. However, PE extract supplementation produced a significant reduction in percentage body fat at the midpoint of the study from day 0–14 (*p* = 0.002) and at the completion of the supplementation period from day 0–28 (*p* = 0.002) (Table 2). A similar trend was observed in fat mass from day 0–14 (*p* = 0.003) and from day 0–28 (*p* = 0.005). In addition, a significant increase in lean body mass from day 0–28 was observed (*p* = 0.025) after the PE treatment. In the placebo group, there were no statistically significant changes in percentage body fat, fat mass, and lean body mass throughout the intervention period (Table 2).

In relation to BP and PWV (Table 2), PE extract produced a significant reduction in SBP from day 0–14 and between day 0–28 (*p* = 0.043, *p* = 0.014, respectively), and DBP from day 0–14 (*p* = 0.046) and between day 0–28 (*p* = 0.009). Statistically significant reductions in PWV (*p* = 0.039, *p* = 0.013) were also obtained in the PE group from day 0–14 and day 0–28. In comparison, there were no significant changes in the placebo group in SBP, DBP and PWV from day 0–28 (Table 2).

### 3.3. Changes of Cortisol and Cortisone Levels in Salivary and Urine Samples

The daily circadian rhythms of salivary cortisol and cortisone in the PE and placebo groups are shown in Table 3. There was a statistically significant reduction in the morning cortisol levels from day 0–14 (*p* = 0.045) and 0–28 in the PE group (*p* = 0.002), but not in cortisone levels. Significant changes in subjects treated with placebo were not observed.

The interconversion of the inert cortisone to active cortisol is catalyzed by the microsomal enzyme 11β-HSD1 and thereby acts as a key for regulating the access of glucocorticoid hormones to the glucocorticoid receptor [41]. There were significant reductions of salivary cortisol/cortisone ratios which indicate 11-β HSD1 activity in the PE group only from day 0 to day 28 at all-time points: morning from a mean of 1.12 ± 0.31 to 0.81 ± 0.33 (*p* = 0.003), noon from 1.31 ± 0.25 to 1.02 ± 0.38 (*p* = 0.047) and evening from 1.39 ± 0.41 to 1.06 ± 0.46 (*p* = 0.045). Changes in the placebo group were not significant.

In 24-h urinary samples, free cortisol excretion was significantly reduced on day 14 as compared with baseline in the PE group (*p* = 0.003), and on day 28 (*p* = 0.043). Other comparisons of cortisol and cortisone levels as well as cortisol/cortisone ratio at 14 and 28 days versus baseline in either the PE or the placebo groups were not statistically significant (Table 4). There was a significant difference between the PE and placebo groups at day 28. Cortisol excretion was lower in the PE group than the placebo (*p* = 0.015), and cortisol/cortisone ratio was also significantly lower in the PE group (*p* = 0.05).

### 3.4. Total Phenolic Compounds, Antioxidant Capacity and Lipid Peroxidation

**Figure 2:**
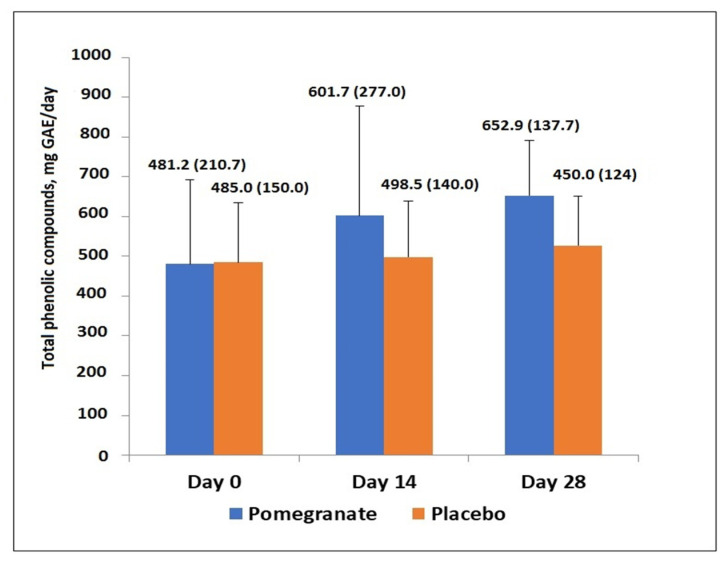
Total phenolic content of 24-h urine samples from days 0 to 28 in both study groups. Increases at days 14 and 28 as compared with baseline were significant in the pomegranate group (*p* = 0.01 at day 14 and *p* = 0.02 at day 28). Between groups, total phenols increase in the PE group at day 28 was significant, *p* = 0.05).

As shown in Figure 2, in the PE group, urinary excretion of total phenolic compounds showed a significant increase at days 14 and 28, from a mean of 481 ± 210.7 to 601.7 ± 277.0 mg/GAE/day (*p* < 0.02) and to 652.9 ± 137.7 mg GAE/day (*p* = 0.02), respectively. Changes of total phenolic compounds in the placebo group were not significant. The increase in total phenols in the PE group at day 28 compared with the placebo was significant (*p* = 0.05).

**Figure 3:**
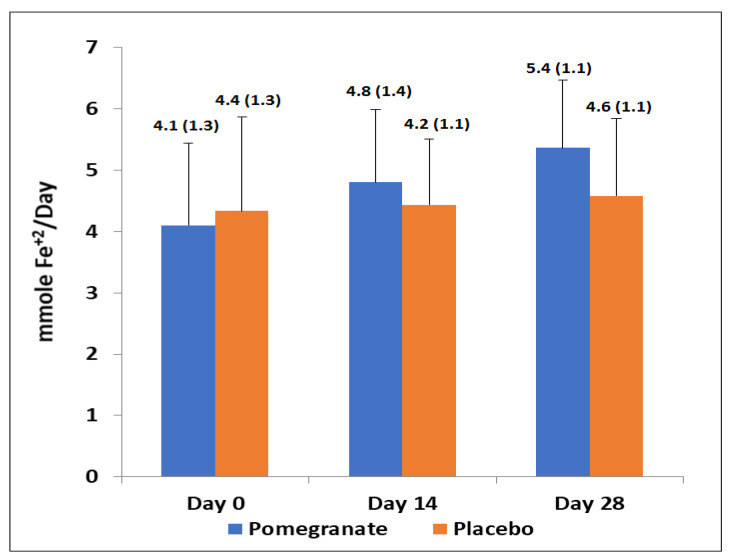
Antioxidant capacity of urine samples from 0–28 days in both study groups. In the pomegranate group, increases from day 0 to day 14 (*p* = 0.01) and day 28 (*p* = 0.02) were significant. Between groups, FRAP increase in the PE group at day 28 was significant, *p* = 0.05).

In relation to total antioxidant capacity (FRAP assay), there was a significant increase after supplementation with PE as compared with baseline values, at 14 days (mean 4.1 ± 1.3 to 4.81 ± 1.4 mmol Fe^+2^/day; *p* = 0.01) and 28 days (5.4 ± 1.1 mmol Fe^+2^/day; *p* = 0.02) (Figure 3), whereas significant changes in the placebo group were not observed. The increase in FRAP in the PE group at day 28 compared with the placebo was significant (*p* = 0.05). There was a strong correlation between urinary excretion of total phenolic compounds and antioxidant capacity in urine samples (*r* = 0.99, *p* < 0.001).

Lipid peroxidation of urine samples was determined using the TBARS assay. There were no significant differences at day 0, 14 and day 28 between the two study groups. There was a slight increase but not significant difference between levels at day 14 in the placebo group (mean levels went up from 2.4 ± 0.3 to 3.2 ± 0.6 µmol MDA/day). However, lipid peroxidation levels remained relatively consistent in the PE group, and there was a slight decrease in TBARS assay values but was not significant (mean levels decreased from 2.46 ± 0.27 to 2.24 ± 0.3 µmol MDA/day at day 14, and 2.16 ± 0.32 µmol MDA/day at day 28). This could be due to the healthy and young type of volunteers.

### 3.5. Bioavailability of Urolithin A Glucuronide

**Figure 4:**
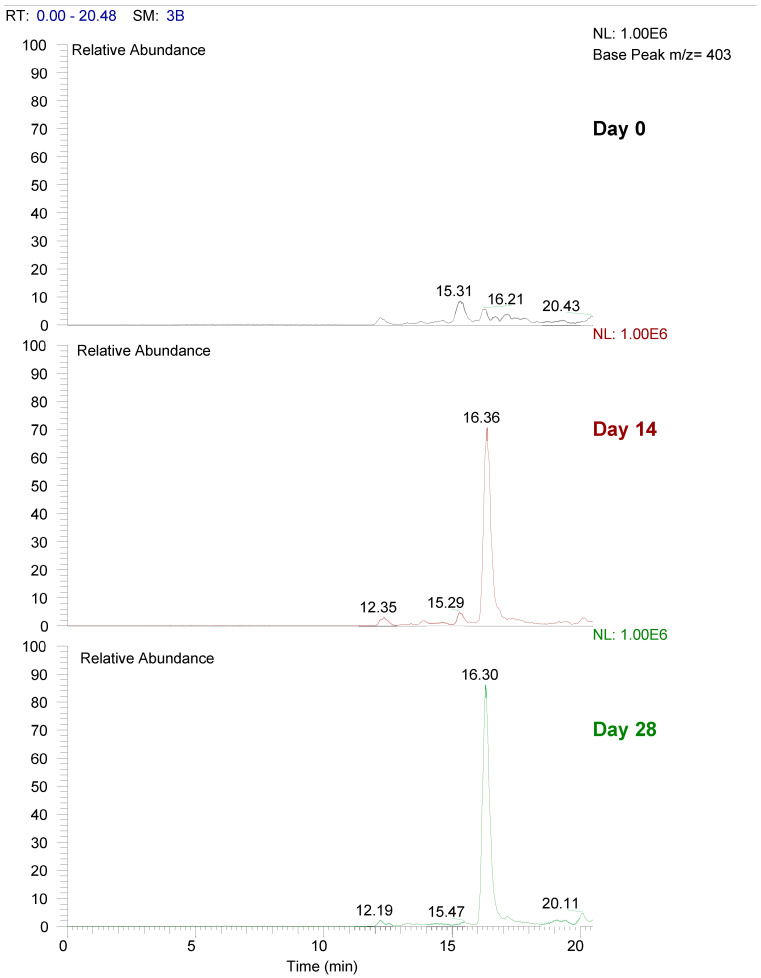
Bioavailability of urolithin A glucuronide in urine samples of one participant assigned to the PE group, at baseline and at days 14 and 28 after consumption of PE.

Urolithin A-glucuronide, a metabolite of ellagic acid was present in the urine on days 14 and day 28 after supplementation with PE, whereas this metabolite was absent before consumption of PE at baseline (see Figure 4).

### 3.6. Nutritional Intake

Assessment of 3-day diet diaries at day 14 of supplementation with PE or placebo was carried out in 12 participants from each group. Similar mean values of energy, protein and fat intake were observed in the two study groups, although the consumption of carbohydrates was significantly higher in the placebo group (Table 5).

## 4. Discussion

Consumption of a natural extract of PE for 28 days was associated with improvements in body composition, decrease in BP, inhibition of salivary cortisol/cortisone ratios (11-β HSD1 activity), reduced cortisol levels in 24-h urine samples, increased in total antioxidant capacity, and slight non-significant increase in lipid peroxidation. All these findings support the anti-hypertensive and beneficial cardiovascular effects of PE, which are consistent with results of previous studies [30,31,42,43].

No significant changes in weight or BMI were observed in both the PE and the placebo groups throughout the study period. However, there was a non-significant slight reduction of body weight. In a 4-week randomized placebo-controlled and cross-over study, consumption of 500 mL of PE juice resulted in a decrease of body weight, although differences at the end of the study as compared with baseline were not statistically significant [30]. Although changes in BMI were not observed, PE supplementation resulted in a significant reduction in the percentage body fat and fat mass in addition to a significant increase in lean body mass. These protective cardiovascular effects are noteworthy given the important endocrine function of adipose tissue and the adverse consequences of adipose tissue excess in relation to hypertension, insulin resistance, dyslipidemia and prothrombotic and proinflammatory states [44].

Decreasing energy intake and the intestinal absorption of dietary fats by inhibiting pancreatic lipase together with the antioxidative and anti-inflammatory effects of the different constituents of PE have been proposed as mechanisms involved in the anti-atherogenic and anti-obesity effects of PE [23]. Providing food with a high antioxidant capacity, thermogenic ingredients and a hypocaloric diet is fundamental to reduce fat mass. In a 16-week, double-blind, randomized, placebo-controlled study that investigated the effects of Xanthigen^®^, a product combining fucoxanthin with punicic acid from pomegranate seed oil, Xanthigen^®^ promoted weight loss, reduced body and liver fat content, and improved liver function tests in obese non-diabetic premenopausal women [45]. However, there is limited data of studies assessing the effects of PE supplementation on body composition in healthy volunteers, and most of the research has been carried out in animal models with results comparable to those obtained in our study. A 12-week study on male C57Bl/J6 mice fed 1 g PE seed oil per 100 g of high fat diet resulted in a reduction in both fat mass and body weight when compared to the control group [46]. In another study in obese rats, PE vinegar contributed to the attenuation of adiposity and increased fatty acid oxidation in the liver [47]. Evidence has found that polyphenols play a role in inhibiting pancreatic lipase activity, influencing fat digestion and energy intake. McDougall et al. [48] found that ellagitannins present in berries could play a role in inhibiting pancreatic lipase activity. Different studies have indicated that PE supplements encompass significant levels of anthocyanins and ellagitannins which may play a role in both fat digestion and excretion [30]. This might explain the reduction in percentage body fat and fat mass observed in this current study since 15 participants had a BMI within the healthy range and 9 were classified as overweight. Further investigations on the mechanisms and the effects of PE supplementation and body composition in non-obese individuals are warranted.

We found that PE supplementation was associated with a significant decrease of SBP and DPB, which is as an important benefit for cardiovascular health. The hypotensive effect of PE has been already demonstrated in previous studies in non-hypertensive and hypertensive subjects [24,25,29,30,31,49,50]. Moreover, PE supplementation caused a significant reduction in PWV. Reductions in PWV may contribute to prevent age-associated arterial stiffness and cardiovascular risk [51]. It has been shown that elevated aortic PWV in healthy community-dwelling older adults as a marker of arterial stiffness was associated with higher cardiovascular mortality, coronary heart disease and stroke [52]. In animal studies, the administration of PE exerted beneficial effects on vascular function and inflammation has been also reported [53]. Interestingly, in a 3-year study in atherosclerotic patients with carotid artery stenosis, consumption of PE juice decreased carotid intima-media thickness and SBP [26]. Such beneficial effects of PE on both BP and PWV can be attributed to the antioxidant and anti-inflammatory properties of their phytochemicals, such as urolithins. We also demonstrated the bioavailability of the metabolite urolithin A glucuronide at days 14 and 28 in a subject given PE. On the other hand, PE supplementation has a positive influence on arterial reactivity, vascular expression of endothelial nitric oxide (NO) synthase and plasma nitrate and nitrite levels [49]. PE has the potential to enhance NO synthase which further relates to the positive effect of reducing BP [29]. The mechanism by which PE consumption reduces BP could also be secondary to its anti-inflammatory and antioxidant activity, enhancing the availability of the vasodilator NO thereby affecting endothelial system and potentially inhibiting angiotensin-converting enzyme (ACE) activity, a key component in the renin angiotensin aldosterone system which regulates BP [54].

Salivary cortisol is thought to provide a more sensitive approach for the assessment of subtle activation of the hypothalamic-pituitary adrenal axis when compared to urine free corticosteroids [55]. Following PE supplementation for 28 consecutive days, there was a clear daily circadian rhythm of salivary cortisol and cortisone, with statistically significant reduction in the morning salivary cortisol levels at day 28 in the PE group. There was also a significant reduction of salivary cortisol/cortisone ratios. Urinary free cortisol exertion was significantly reduced at day 14 and day 28 following PE intake, and there were no significant changes in urinary free cortisone and cortisol/cortisone ratios. In the placebo group, significant differences in salivary or urinary glucocorticoids were not found. In the study of Tsang et al. [30], PE juice supplementation led to reductions in BP which was likely mediated partly through the inhibition of 11-β HSD 1 enzyme activity as evidenced by a reduction in cortisol/cortisone ratio. Furthermore, in a previous randomized double-blind controlled study in healthy volunteers, a significant reduction in both salivary cortisol and cortisol/cortisone ratio following a 4-week treatment with PE was observed [24].

Supplementation with PE was associated with a marked enhancement of antioxidant activity as shown by an increase in total antioxidant capacity (FRAP assay) and total phenolic compounds, with no significant changes in the placebo group. The antioxidant capacity of PE has been previously shown in other investigations [24,29,30]. Although participants were requested to reduce their intake of foods rich in polyphenols this was not quantified and compliance with this recommendation was not evaluated. We found a strong correlation between total phenolic compounds and the antioxidant capacity. This suggested that total phenolics exertion can significantly contribute to most of the antioxidant capacity of the urine. This was consistent with previous investigations that found the antioxidant activity of PE juice was largely accountable to ellagic acid derivatives, anthocyanins, and hydrolysable tannins [56]. The antioxidant and anti-inflammatory mechanisms of polyphenols are thought to be multifactorial. Polyphenols exert antioxidant effects through the increase in activity of antioxidant enzymes, the inhibition of lipid peroxidation, the scavenging of free radicals [57], and reduce oxidation via the chelation of metal ions [57,58]. Moreover, Mediterranean diet rich in polyphenols can influence several signaling pathways (nicotinamide adenine dinucleotide phosphate oxidase), NF-κB-mediated oxidative stress (Nuclear factor kappa-light-chain-enhancer of activated B cells), and metabolic inflammation [59], and improves urinary inflammation and oxidation parameters [60]. The relationship of antioxidant and anti-inflammatory effects of polyphenols for protection in CVD is evident via the mechanisms mentioned above and molecular mechanisms that mediate for example obesity-related oxidative stress and inflammation [61].

TBARS is an essential biomarker of oxidative stress as it measures the damaging products of lipid oxidation found in the urine [62]. In our study, no significant changes in lipid peroxidation were observed in either group throughout this intervention. Nonetheless, TBARS levels appeared to increase in the placebo group whereas levels were reduced in the PE group, a likely effect of PE supplementation. Therefore, the role of PE supplementation on lipid peroxidation may be better assessed before and after exercise, but exercise testing was not included in the present design.

Punicalagin is one of the most predominant ellagitannin found in PE and is largely accountable for its potent antioxidant activity [63,64]. In both animal and human models, ellagitannins as well as ellagic acid have demonstrated relevant biological, anti-inflammatory, antioxidant, anti-carcinogenic effects suggesting potential preventative mechanisms against diabetes, cancer, neurological and cardiovascular diseases [62,65]. However, ellagitannins and ellagic acid have a poor absorption due to lower water solubility of free ellagic acid, ionization at physiological pH to form poorly soluble complexes with calcium and magnesium ions in the intestine, and the ability to bind to the intestinal epithelium [63]. Moreover, gut microbiota in the colon is further involved to metabolize the unabsorbed compounds to urolithins, suggesting that urolithins may be the actual bioactive molecules [66]. A study investigating the bioavailability of PE ellagitannins found that consumption of pomegranate resulted in the detection of urolithins A, B and C in both the plasma and urine of rats [67]. Due to time constrains, it was not feasible to carry LC-MS analysis on all participants in this study and thus a urine sample of a randomly selected PE-treated subject was chosen. The results obtained clearly showed the presence of urolithin A-glucuronide only at day 14 and 28 following PE intake and indicate that LC-MC analysis is a useful method of determining the bioavailability of PE polyphenols. However, it is possible that similar results may not have been observed in all participants due to inter-individual variability and differences in microbiota composition.

Several researchers have reported that antioxidant supplements may be used to improve athletic performance. We have previously investigated the effects of natural pomegranate juice intake on exercise performance and oxidative stress and found that pomegranate juice attenuates exercise-induced oxidative stress and blood pressure and increases the distance travelled [67]. However, we do not think that the type and workload might have influenced the variables measured since all participants were refrained from exercising at least 24 h before the measurements. Recently, Sellitto et al. [68] reported that antioxidant supplementation may hinder the Role of exercise training as a natural activator of sirtuin 1 (SIRT1) which is activated by polyphenols antioxidants. On the other hand, highly trained endurance athletes did not benefit from consuming nitrate-rich foods but might have benefited from polyphenol consumption [69].

The main limitation of the study was the small, considerably young sample size, all of which were students. Another issue was the inability to control the diet of participants and day-to-day fluctuations in dietary intake made it problematic to investigate the true effect of pomegranate extract on anthropometric and physiological makers. Additionally, the possibility that participants may not have maintained their regular diet and exercise regime throughout the intervention. Furthermore, while the use of diet diaries provided a glimpse into the dietary habits of the participants, pre and post diet diaries would have been more beneficial to determine alterations in dietary habits throughout the intervention. Macronutrients were only quantified, and overall phenolic content of the diet was not quantified. Perhaps assessing polyphenol intake using food frequency questionnaires would have proved beneficial.

## 5. Conclusions

This study showed that a natural PE extract possesses antioxidant property that attenuated oxidative stress and caused an increase in urinary antioxidant capacity and total phenolic products. Furthermore, following 28-day PE supplementation, metabolites of PE polyphenols were present in urinary samples. The PE extract possesses anti-hypertensive, anti-atherogenic and cardioprotective effects and highlights the beneficial effects of PE on BP, arterial stiffness, and body composition, all of which are potential risk factors in the development of cardiovascular disease. Continued research in relation to pomegranate polyphenols is fundamental to determine the optimal dose of polyphenols necessary to impart beneficial biological effects. It would be of great interest to study the effects of PE in certain groups at risk for cardiovascular events (i.e., diabetics, dyslipidemics, hypertensives, etc.), and the high-risk subgroups for cardiovascular disease. Additionally, the exploration of the effect of pomegranate extract on a range of alternative parameters that may be manipulated by its unique combination of polyphenols should be further investigated in a larger scale trial to shed light on this topic.

## Figures and Tables

**Table 1:** Baseline/pre-treatment (day 0) characteristics of participants and study variables.

Variables	Pomegranate (*n* = 12)	Placebo (*n* = 12)	*p*-Value
Age, years	23.5 ± 1.8	24 ± 2.4	0.632
Body weight, kg	69.8 ± 14.6	71.6 ± 14.1	0.695
Height, cm	1.7 ± 0.1	1.7 ± 0.2	0.960
BMI, kg/m^2^	24.1 ± 2.7	24.7 ± 2.9	0.446
Fat mass, %	24.2 ± 5.0	23.2 ± 8.5	0.680
Fat mass, kg	16.4 ± 2.9	15.8 ± 5.1	0.875
Lean mass, kg	54.4 ± 14.1	55.1 ± 15.2	0.364
SBP, mmHg	118.1 ± 6.6	118.2 ± 11.5	0.854
DBP, mmHg	68.9 ± 5.1	71.2 ± 7.2	0.750
PWV, m/s	6.5 ± 0.6	6.5 ± 0.5	0.763
Cortisol, urinary, nmol/day	125.2 ± 63.1	112.6 ± 99.2	0.481
Cortisone, urinary, nmol/day	109.7 ± 48.7	96.6 ± 30.7	0.355
Cortisol/cortisone ratio, urinary	1.14 ± 0.68	1.16 ± 0.75	0.729
Cortisol, salivary, ng/mL	5.66 ± 1.3	4.996 ± 3.5	0.283
Cortisone, salivary, ng/mL	4.83 ± 1.47	4.649 ± 3.1	0.315
Cortisol/cortisone ratio, salivary	1.22 ± 0.27	1.075 ± 0.4	0.089
Total phenolics, mg GAE/day	436.2 ± 94.9	430.1 ± 236.3	0.293
Antioxidant capacity, FRAP, mmol Fe^+2^/day	4.7 ± 1.2	5.2 ± 1.9	0.285
Lipid peroxidation, TBARS, µmoL MDA/day	2.46 ± 0.27	2.4 ± 0.3	0.543

Data expressed as mean ± SD. BMI: body mass index; SBP: systolic blood pressure; DBP: diastolic blood pressure; PWV: pulse wave velocity; GAE: gallic acid equivalent; FRAP: ferric reducing antioxidant power; TBARS: thiobarbituric acid reactive substances; MDA: malondialdehyde. Comparisons between PE and placebo were made using independent *t*-tests.

**Table 2:** Changes of anthropometric, body composition and BP variables in the study groups.

Variables	Pomegranate (*n* = 12)			Placebo (*n* = 12)		
	Day 0	Day 28	*p*-Value	Day 0	Day 28	*p*-Value
Weight, kg	70.9 ± 14	70.6 ± 13.9	0.170	71.6 ± 13.9	71.5 ± 14.2	0.977
BMI, kg/m^2^	23.6 ± 2.9	23.4 ± 2.9	0.157	24.7 ± 2.9	24.7 ± 3.1	0.848
Fat mass, %	24.1 ± 4.1	23.5 ± 3.8	0.002	23.2 ± 8.5	22.8 ± 8.4	0.069
Fat mass, kg	16.4 ± 2.9	15.7 ± 2.9	0.005	15.8 ± 5.1	15.0 ± 4.8	0.119
Lean mass, kg	54.4 ± 14.1	55.8 ± 13.9	0.025	55.1 ± 14.9	55.5 ± 15.6	0.075
SBP, mmHg	118.1 ± 6.6	113.9 ± 9.4	0.014	118.2 ± 11.5	116.8 ± 11.2	0.444
DBP, mmHg	68.9 ± 5.1	65.2 ± 6.1	0.009	71.2 ± 7.2	71.7 ± 6.9	0.756
PWV, m/s	6.6 ± 0.5	6.1 ± 0.5	0.013	6.5 ± 0.5	6.4 ± 0.4	0.203

Data expressed as mean ± SD. BMI: body mass index; SBP: systolic blood pressure; DBP: diastolic blood pressure; PWV: pulse wave velocity. Comparisons between PE and placebo were made using independent *t*-tests.

**Table 3:** Changes of salivary cortisol and cortisone levels in the two study groups.

Study Groups and Time	Study Day	Cortisol, ng/mL	*p*-Value	Cortisone, ng/mL	*p*-Value
Pomegranate					
Morning	Day 0	8.02 ± 2.6		7.5 ± 2.8	
	Day 14	6.42 ± 1.1	0.045	6.96 ± 2.4	0.35
	Day 28	5.65 ± 1.9	0.002	8.11 ± 4.2	0.58
Noon	Day 0	5.39 ± 0.8		4.33 ± 1.2	
	Day 14	4.57 ± 1.3	0.07	4.34 ± 1.2	0.98
	Day 28	4.51 ± 1.9	0.12	6.08 ± 2.9	0.38
Evening	Day 0	3.6 ± 1.4		2.65 ± 1.1	
	Day 14	3.45 ± 1.5	0.82	2.93 ± 0.8	0.48
	Day 28	3.36 ± 1.8	0.61	3.26 ± 2.9	0.23
Placebo					
Morning	Day 0	7.15 ± 3.5		6.848 ± 3.1	
	Day 14	7.28 ± 3.5	0.456	7.025 ± 3.8	0.186
	Day 28	8.11 ± 2.9	0.340	6.435 ± 3.5	0.886
Noon	Day 0	4.66 ± 3.3		3.88 ± 0.8	
	Day 14	4.59 ± 1.8	0.524	4.16 ± 1.6	0.247
	Day 28	8.11 ± 3.6	0.752	4.734 ± 2.2	0.343
Evening	Day 0	3.17 ± 3.9		3.22 ± 2.4	
	Day 14	3.24 ± 1.9	0.758	3.027 ± 2.6	0.394
	Day 28	3.54 ± 1.5	0.794	3.384 ± 1.2	0.859

Data expressed as mean ± SD. Comparisons were made using *t*-tests for paired samples.

**Table 4:** Urinary excretion of cortisol and cortisone in the two study groups.

Study Day	Pomegranate Group (*n* = 12)			Placebo Group (*n* = 12)		
	Cortisol nmol/Day	Cortisone nmol/Day	Cortisol/ Cortisone Ratio	Cortisol nmol/Day	Cortisone nmol/Day	Cortisol/ Cortisone Ratio
Day 0	125.9 ± 63.1	109.7 ± 48.8	1.14 ± 0.68	122.6 ± 99.6	96.4 ± 30.7	1.17 ± 0.85
Day 14	99.87 ± 44.9	111.6 ± 39.9	0.89 ± 0.47	128.2 ± 99.4	116.1 ± 32.2	1.08 ± 0.82
Day 28	99.1 ± 34.3	109.3 ± 43.3	0.906 ± 0.41	137.7 ± 61.7	110.6 ± 39.3	1.24 ± 1.17
Days 14 vs. 0, *p* value	0.003	0.859	0.243	0.174	0.069	0.953
Days 28 vs. 0, *p* value	0.043	0.716	0.219	0.434	0.433	0.078

Between PE and placebo groups: At day 28, significant Cortisol excretion (*p* = 0.015), and Cortisol/cortisone ratio. (*p* = 0.05). Data expressed as mean ± SD. Comparisons were made using *t*-tests for paired samples.

**Table 5:** Energy and macronutrient consumption in the PE and placebo groups on day 14.

Variables	Pomegranate (*n* = 12)	Placebo (*n* = 12)	*p* Value
Energy, kcal	1884.8 ± 741.2	2052.9 ± 825.1	0.467
Proteins, g	109.9 ± 64.7	107.9 ± 74.8	0.921
Carbohydrates, g	196.4 ± 53.9	223.7 ± 81.1	<0.05
Fat, g	76.4 ± 44.4	84.6 ± 35.8	0.272

Data expressed as mean ± SD.

## Data Availability

Data contained within the article are available from the corresponding author upon request.
